# Editorial: Prebiotics in the management of obesity and associated metabolic disorders

**DOI:** 10.3389/fendo.2024.1432530

**Published:** 2024-07-03

**Authors:** Sepehr Khosravi, Ozra Tabatabaei-Malazy, Solaleh Emamgholipour, Mozhdeh Sojoodi, Parisa Shabani

**Affiliations:** ^1^ Non-Communicable Diseases Research Center, Endocrinology and Metabolism Population Sciences Institute, Tehran University of Medical Sciences, Tehran, Iran; ^2^ Endocrinology and Metabolism Research Center, Endocrinology and Metabolism Clinical Sciences Institute, Tehran University of Medical Sciences, Tehran, Iran; ^3^ Department of Clinical Biochemistry, School of Medicine, Tehran University of Medical Sciences, Tehran, Iran; ^4^ Metabolic Disorders Research Center, Endocrinology and Metabolism Molecular-Cellular Sciences Institute, Tehran University of Medical Sciences, Tehran, Iran; ^5^ Division of Surgical Oncology, Massachusetts General Hospital, Harvard Medical School, Boston, MA, United States; ^6^ Frankel Institute For Heart and Brain Health, University of Michigan, Medial Center, Ann Arbor, MI, United States

**Keywords:** weight management, overweight, obesity, microbiota, weight regain, prebiotics

The prevalence of obesity and metabolic comorbidities has considerably increased worldwide over the past decades (1). Extensive efforts have been made to address obesity, but current therapeutic approaches have not yielded satisfactory results, indicating a need for alternative solutions. Imbalanced gut microbiota or dysbiosis is thought to be a principal factor in the development of obesity and metabolic consequences. The approach of modulating the gut microbiota using oral supplementation with prebiotics has received the lion’s share of attention for its beneficial effects on managing obesity and associated metabolic disorders. Preclinical and clinical studies have reported the benefits of prebiotics in combatting obesity and metabolic abnormalities through various mechanisms of action. However, the results are inconsistent, necessitating further studies to validate the conclusions. Further detailed studies are needed to understand how changes in microbial signatures affect the management of obesity and its related conditions, such as diabetes and fatty liver. This editorial on the research topic “Prebiotics in the Management of Obesity and Associated Metabolic Disorders” explores the therapeutic potential of modifying gut microbiota through various means, including diet, prebiotics, probiotics, and synbiotics. It highlights innovative strategies that could lead to significant advances in the management and treatment of metabolic disorders.

The burgeoning field of gut microbiota research offers novel insights into how our internal microbial communities affect our health, particularly about metabolic diseases such as obesity and nonalcoholic steatohepatitis (NASH). With obesity reaching epidemic proportions globally and NASH becoming more recognized as a major cause of liver disease, understanding the role of gut microbiota in these conditions is becoming crucial (1). Xiang et al. study delves into how the approximately 40 trillion microbes in our gut can influence NASH progression. It highlights the critical role of microbial dysbiosis in altering hepatic lipid metabolism and inflammatory responses, pointing out that microbial metabolites, especially short-chain fatty acids (SCFAs) derived from dietary fibers, are vital. SCFAs like butyrate provide substantial energy to intestinal cells and have profound effects on intestinal and liver health (2, 3). It is well known that gut microbiota plays a key role in the metabolism of bile acids in liver homeostasis, facilitated by receptors such as the farnesoid X receptor (FXR) and Takeda G protein-coupled receptor 5 (TGR5) (4). Insufficient FXR and TGR5 activities under NASH conditions highlight the need to develop microbiota-centered therapies that could transform the treatment landscape for liver diseases by leveraging microbial interactions. Xiang et al. emphasize that the relationship between gut microbiota and NASH remains complex and requires further studies to establish a clear roadmap for the clinical application of microbiota.

Dietary treatment is recognized for its anti-obesity effects by altering gut microbiota composition (5, 6). Hence, it highlights a novel therapeutic strategy that could be pivotal in managing obesity and its associated metabolic complexities. Song et al. study explores the anti-obesity effects of Platycodon Grandiflorum Polysaccharide (PGNP) by gut microbiota modulation. This intervention has shown promising results in rebalancing gut microbial communities, notably increasing Bacteroidetes and reducing Firmicutes. These changes are associated with enhanced production of metabolites that positively influence lipid metabolism and inflammation—key players in obesity and metabolic syndrome.

Furthermore, polyamine metabolites, essential for cellular health and immune function, can be synthesized by gut microbiota from amino acids and regulated by host cells, gut microbiota, and diet (7). The potential of polyamine synthesis enhancement in treating obesity-related type 2 diabetes and metabolic syndrome is gaining attention in the study of Bui et al. Increasing polyamine levels through diet and probiotic supplementation may mitigate the immune deficiencies and metabolic disruptions accompanying these conditions. This approach underscores the importance of gut microbiota in metabolic health and opens up new avenues for creating nutraceutical interventions that capitalize on these mechanisms.

Child obesity is another area of research focus, as this group requires further attention due to the long-term effects of obesity. Prebiotics may help fight childhood obesity by altering gut bacteria, which is a promising research area. Despite the lack of extensive clinical trials, preclinical evidence suggests that prebiotics can significantly influence host metabolism. Wang et al. highlight the importance of further research on how prebiotics work and developing focused treatments to help reduce rising childhood obesity rates. There are several key mechanisms, including the modulation of enteroendocrine function and the systemic effects of prebiotics and their fermentation products on lipid and glucose homeostasis (8).

Lastly, Rasaei et al. study shows the overall effectiveness of biotics—prebiotics, probiotics, and synbiotics—across various demographics. They found an inverse association between biotics consumption and overweight/obesity risk in adults. Moreover, the consumption of prebiotic: 8-66 g/day, probiotic: 10 4 -1.35×10^15^ colony-forming unit (CFU)/day, and synbiotic: 10 6 -1.5×10^11^ CFU/day and 0.5-300 g/day for 2 to 104 weeks showed a favorable effect on the overweight/obesity indicators in adults. However, while generally effective in reducing obesity-related measures such as body weight and BMI in adults, their impact is less significant in pregnant women and infants. Some of the challenges in determining the effectiveness of currently available probiotic preparations for weight control are the differences in the strains and formulations of probiotics used, individual responses to probiotics, dietary habits, genetic predispositions, lifestyle factors, personal gut microbiota composition, intervention duration, specific target population, and the quality of study designs (9). These findings highlight the urgent need for well-designed clinical trials to explore how biotics can influence obesity and metabolic health comprehensively and effectively across diverse demographic groups.

As we unravel the complex interactions between our gut microbiota and metabolic health, targeted interventions focusing on this interplay may offer viable solutions for managing and potentially reversing the effects of metabolic diseases. The studies reviewed here provide a hopeful glimpse into the future of metabolic disorder treatments, suggesting that with further research and clinical trials, we can better tailor these interventions to benefit individuals at various stages of life, from infancy through adulthood. Emphasizing the need for continued research, these insights herald a new era in medical science where gut microbiota modulation becomes a cornerstone of innovative therapeutic strategies.

## Author contributions

SK: Validation, Visualization, Writing – original draft, Writing – review & editing. OT-M: Conceptualization, Supervision, Validation, Visualization, Writing – review & editing, Writing – original draft. SE: Conceptualization, Supervision, Validation, Writing – review & editing. MS: Validation, Visualization, Writing – review & editing. PS: Validation, Visualization, Writing – review & editing.
